# Image-guided intensity-modulated radiotherapy in patients with FIGO IIIC1 cervical cancer: efficacy, toxicity and prognosis

**DOI:** 10.7150/jca.81953

**Published:** 2023-04-09

**Authors:** Qingyu Meng, Xiaoliang Liu, Ke Hu, Xiaorong Hou, Fuquan Zhang, Weiping Wang, Junfang Yan, Bo Yang, Wenbo Li

**Affiliations:** Department of Radiation Oncology, Peking Union Medical College Hospital, Chinese Academy of Medical Sciences & Peking Union Medical College, Beijing, People's Republic of China.

**Keywords:** cervical cancer, FIGO IIIC1, IG-IMRT, chemotherapy, toxicity, prognosis

## Abstract

**Background:** To investigate the efficacy, toxicity and prognosis of image-guided intensity-modulated radiotherapy (IG-IMRT) in patients with FIGO IIIC1 cervical cancer.

**Methods:** We retrospectively reviewed clinical records of patients with FIGO IIIC1 cervical cancer treated with definitive IG-IMRT in our institute from January 2008 to December 2017. A dose of 50.4Gy in 28 fractions was prescribed to at least 95% of PCTV, the positive pelvic lymph nodes received a dose of 56-61.6Gy in 28 fractions with simultaneous integrated boost (SIB). Weekly cone beam compute tomography (CBCT) and daily megavoltage CT (MVCT) was performed before treatment. Both 2D brachytherapy and 3D brachytherapy were allowed in our study. Weekly Cisplatin (30-40mg/m2) was the first line regimen for concurrent chemotherapy. Overall survival (OS), disease free survival (DFS), local control (LC) and local regional control (LRC) was calculated with Kaplan-Meier method. Cox proportional hazard model was used to perform univariate and multivariate analyses.

**Results:** A total of 502 patients were enrolled in this study. The median follow-up duration was 42.1 months (range: 2.3-137.3 months). The 3-year and 5-year estimated OS, DFS, LC, LRC were 81.7% and 75.5%, 71.4% and 68.6%, 89.9% and 89.9%, 86.1% and 84.3%, respectively. The incidences of chronic grade 3 or greater gastrointestinal and genitourinary toxicities were 2.7 % and 0.8%, respectively. Pelvic lymph nodes recurrence occurred in 21 patients (4.2%). Advanced T stage was identified as adverse factor for OS and LC. More positive lymph nodes (≥2) were associated with worse OS, DFS and LRC. The cycles of concurrent chemotherapy significantly affected OS, DFS and LRC.

**Conclusion:** For patients with FIGO IIIC1 cervical cancer, IG-IMRT was well tolerated with excellent survivals. T stage and number of positive lymph nodes significantly influenced the survivals indicating the heterogeneity of stage IIIC1 cervical cancer patients. Adequate cycles of chemotherapy (≥4 cycles) was of great value for this group of patients.

## Background

Pelvic lymph node metastasis (PLNM) is an adverse prognostic factor for patients with cervical cancer, which has been identified in many previous studies 1-4. For this reason, the FIGO 2018 staging system defined patients with pelvic lymph node metastasis (PLNM) as stage IIIC1^5^. The treatment for cervical cancer patients with stage IIIC1 varied based on the primary tumor T stage. For patients with early T stage (FIGO2009 IB1-IIA1), definitive surgery followed by adjuvant chemoradiotherapy (CCRT) and definitive concurrent CCRT are all recommended 6. While Definitive concurrent CCRT is the standard treatment for patients with more advanced T stage (FIGO2009 IIB or more advanced stage) 6. Since the present treatment choices for FIGO IIIC1 cervical cancer were different, studies specially focus on patients with IIIC1 stage were rare, most studies included patients with other stages at the same time 3, 7, 8.

Pelvic lymph node recurrence is a common failure pattern after CCRT. In the EMBRACE study 9, the incidences of lymph node failure were 7% for patients with N- and 16% for patients with N+ before treatment. How to improve the lymph node control rate is an important issue for radiation oncologists to consider. Radiation dose is significantly associated with the lymph node control rate 10, 11. In the time of intensity modulated radiotherapy (IMRT), simultaneous integrated boost (SIB) can deliver different dose level to different target volume at the same time. With this method, we can achieve an increased dose to positive lymph nodes without a prolongation of radiotherapy duration. It can also increase the biological equivalent dose (BED) of positive lymph nodes 10, 12. Furthermore, with the help of image-guided radiotherapy (IGRT), we can make sure that the high dose is delivered to the right target. Previous study has reported the benefit of dose escalated radiotherapy with SIB to positive lymph nodes 10. However, the most effective dose and the high dose related toxicity need to be further discussed 12.

The prognosis of patients with stage IIIC1 cervical cancer is complicated and controversial. After the new FIGO staging system was released, studies based on SEER and NCDB database revealed that stage IIIC1 was a heterogeneous group 13, 14, the T stage affected the survivals significantly 13. Except for T stage, the characteristics of positive lymph node, such as size, number, volume and site 1, 2 were also identified as prognostic factors for stage IIIC1 patients in other studies. Unfortunately, all these studies were just limited to one particular aspect - T stage or lymph nodes. They failed to combine these factors together and drew a consistent conclusion.

In this study, we reported our experience in the treatment of patients with stage IIIC1 cervical cancer with image guided-IMRT (IG-IMRT), specially focusing on the efficacy, toxicity and prognosis.

## Methods

### Patients selected

After the protocol was approved by Institute Review Board (IRB) of Peking Union Medical College Hospital, we retrospectively reviewed clinical records of patients with cervical cancer treated with definitive IG-IMRT between January 2008 and December 2017 at our institute. The inclusion criteria were as follows: histological proven cervical cancer, FIGO IIIC1 stage, scheduled to receive definitive IG-IMRT. The pretreatment evaluation included gynecological examination, complete blood counts, biochemical analysis, squamous cell carcinoma antigen (SccAg), CA125, chest and abdomen compute tomography (CT), pelvic magnetic resonance imaging (MRI) or positron emission tomography (PET)/CT. Positive pelvic lymph node was defined as a minimal diameter of ≥ 10mm on CT imaging or diagnosed by PET/CT.

### Radiotherapy

As described in our previous studies 15, all patients received a CT simulation before treatment (16-slice Philips Brilliance Big Bore CT). An empty rectum and full bladder were prepared before simulation. Oral and intravenous contrast agents were used to help identify small intestines and blood vessels.

All enrolled patients were treated with definitive IMRT including fixed-field IMRT (FF-IMRT), volumetric modulated arc therapy (VMAT) and helical tomotherapy (HT). The clinical target volume (CTV) included the primary tumor, cervix, uterus, parametrium, vagina (depending on the extend of primary tumor) and pelvic lymph node region. For patients with high risk of para-aortic lymph node metastasis, the para-aortic lymph node region was also involved in CTV. Positive pelvic lymph node was defined as gross tumor volume (GTV). Planning clinical target volume (PCTV) included CTV plus a 7-10 mm margin. A 5mm margin was added to GTV to create planning gross tumor volume (PGTV). A dose of 50.4Gy in 28 fractions was prescribed to at least 95% of the PCTV. At least 95% of the PGTV received a dose of 56-61.6Gy in 28 fractions with SIB.

**Figure 1:**
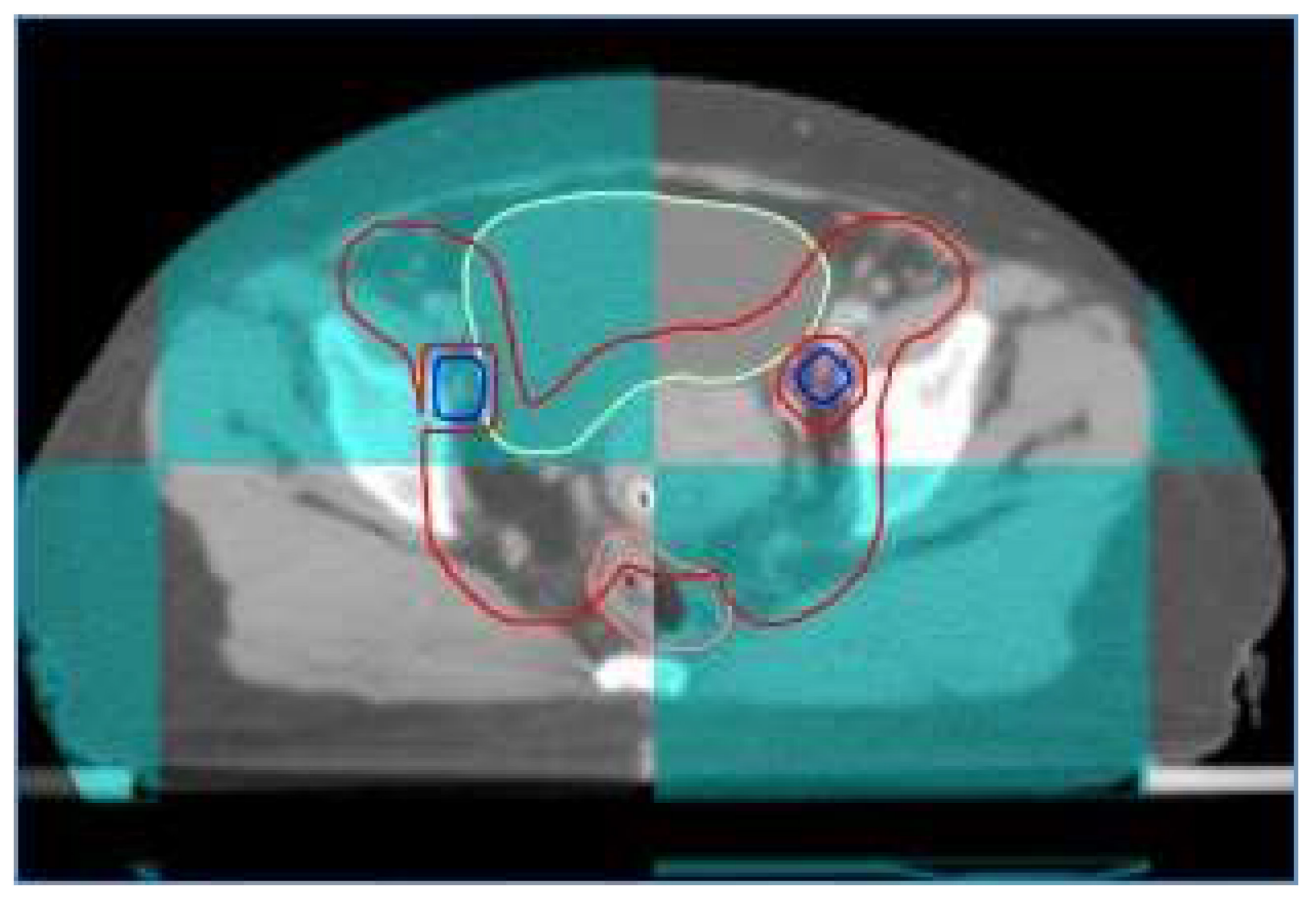
A patient with FIGO IIIC1 cervical cancer treated with IG-IMRT (Cone beam CT).

At our institute, image guide radiotherapy (IGRT) was routinely performed for patients with cervical cancer receiving definitive IMRT. For patients treated with FF-IMRT and VMAT, weekly cone beam compute tomography (CBCT) was performed. Daily on-board megavoltage CT (MVCT) was conducted for patients receiving HT every day before treatment (shown in Figure 1).

High dose rate (HDR) brachytherapy was generally administered after three weeks of external beam radiotherapy. Both 2D and 3D brachytherapy were allowed in our center. A dose of 30-36Gy in 5-6 fraction was prescribed to point A for patients with 2D brachytherapy. If 3D brachytherapy was conducted, at least 90% of the high-risk CTV (HR-CTV) should receive a dose of 30Gy in 5 fractions.

### Chemotherapy

Neoadjuvant and adjuvant chemotherapy were not routinely used in our center. Weekly Cisplatin (30-40mg/m2) was the first line regimen for concurrent chemotherapy, while weekly paclitaxel (60-80mg/m2) was an alternative in patients with renal dysfunction.

### Follow up and toxicity evaluation

All patients received first follow-up examination one month after the treatment, if residual tumor existed, brachytherapy or other adjuvant treatment would be adopted. Then patients received follow up examination every 3 months in the first 2 years, every 6 months during the next 3-5 years, once a year after 5 years. The routine examinations included gynecological examination, SccAg, CA125, chest and abdomen CT, pelvic MRI. PET/CT was not routinely used unless disease relapse were suspected. The Common Terminology Criteria for Adverse Events version 4.0 (CTCAE 4.0) was used for evaluating the treatment related toxicities.

### Statistical analyses

Overall survival (OS) was defined as interval between the date of the start of the treatment and the date of death or last follow-up. Disease free survival (DFS) was counted from the beginning of the treatment to the date of disease recurrence or last follow-up. Local control (LC) referred to the time between the beginning of the treatment and the date of the primary tumor relapse or last follow up. The time from the start of treatment to the date of pelvic recurrence or last follow up was regarded as local regional control (LRC).

OS, DFS, LC and LRC were calculated with Kaplan-Meier method, univariate and multivariate analyses were performed with cox proportional hazard model. SPSS 23.0 software (SPSS Inc, Chicago, IL, USA) was used to perform all statistical analyses, a two-side value of P<0.05 was regarded as statistically significant.

## Results

### Patients and treatment characteristics

A total of 502 patients were enrolled in this study. The median age was 50 years old (range: 26-78 years old). Squamous cell carcinoma was the primary histological type, accounting for 93.8% of all patients. Most patients were classified into T2 stage (353/502, 70.1%), the T1 and T3 stage included 50 (10.0%) and 100 (19.9%) patients, respectively. 285 patients (56.8%) had two or more positive lymph nodes, while 177 patients (35.2%) had only one pelvic lymph node metastasis. Based on the location of positive lymph nodes, we divided patients into two groups (single region LNM 321 patients, multi regions LNM 141 patients). More than 65% of patients received a dose escalation of ≥60Gy to the positive lymph nodes. Due to the retrospective nature, 40 patients' detailed LNM message were missing. Only two patients did not complete the radiotherapy procedure, they received surgery in other hospitals after 20 fractions of external radiotherapy and one fraction of brachytherapy. Concurrent chemotherapy was performed in 466 patients (92.8%), and 407 patients (81.1%) received 4 or more cycles of chemotherapy. 17 patients (3.4%) received adjuvant chemotherapy after CCRT. The detailed information of patients and treatment characteristics were listed in Table 1.

### Survival outcomes

**Figure 2:**
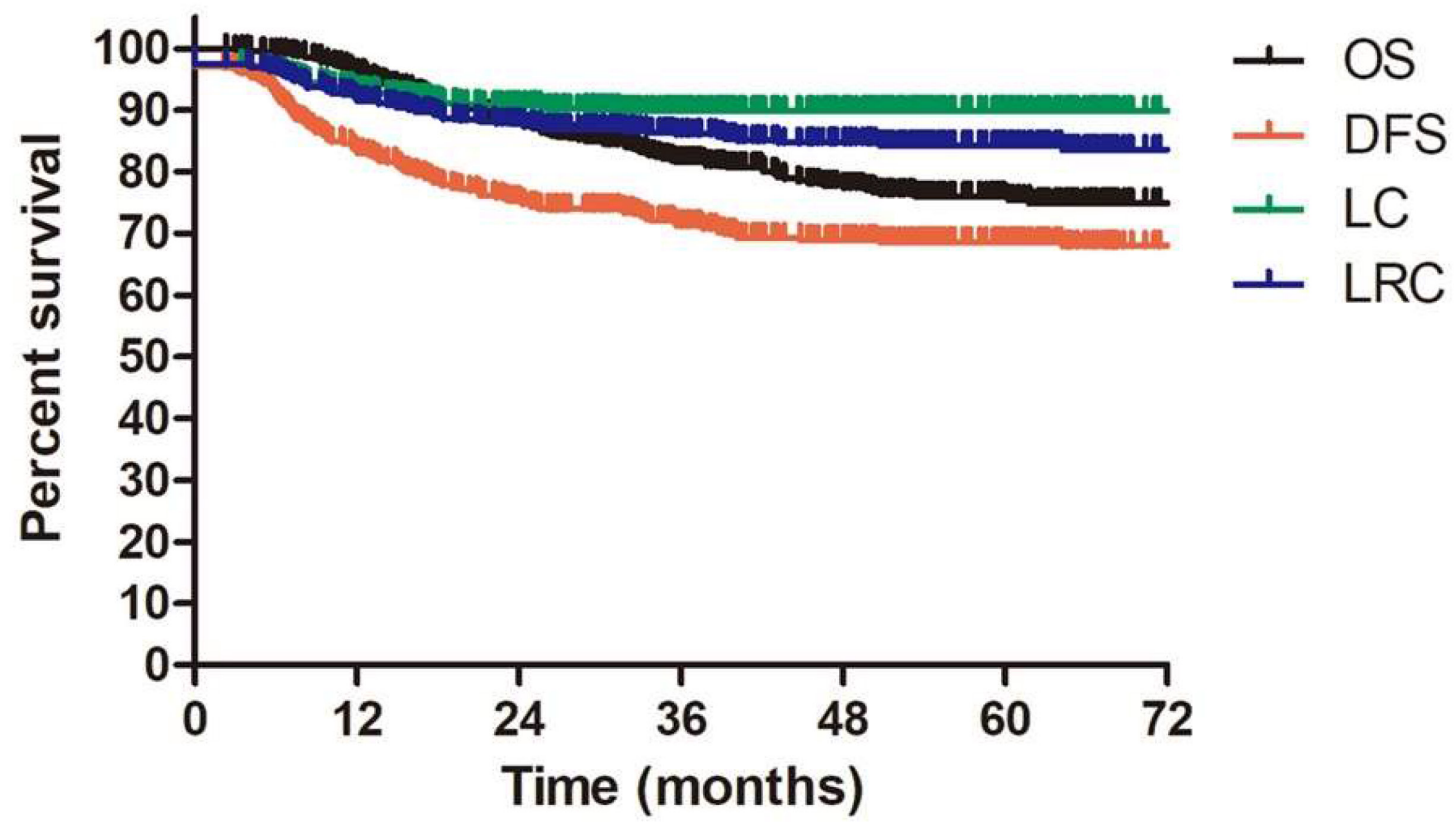
OS, DFS, LC, LRC for patients with IIIC1 cervical cancer

The median follow-up duration for all enrolled patients and alive patients were 42.1 months (range: 2.3-137.3 months) and 52.1 months (range: 2.3-137.3 months). 144 patients underwent more than 60 months follow up. The 3-year OS, DFS, LC and LRC were 81.7%, 71.4%, 89.9% and 86.1%, respectively. The estimated 5-year OS, DFS, LC and LRC were 75.5%, 68.6%, 89.9% and 84.3%, respectively (Figure 2).

### Disease failure patterns

As shown in Table 2, a total of 151 patients (30.1%) experienced disease failure during follow up, including 68 patients (13.5%) with pelvic recurrence and 101 patients (20.1%) with distant metastasis. Eighteen patients (3.6%) suffered both pelvic relapse and distant failure at the same time. Among patients who developed pelvic recurrence, thirteen patients (2.6%) had persistent disease. The other most common sites of recurrence included cervix (19/502, 3.8%), pelvic lymph node (21/502, 4.2%), parametrium (8/502, 1.6%), uterus (4/502, 0.8%) and vagina (4/502, 0.8%). For 134 patients receiving < 60Gy irradiation to PLNM, five patients (3.7%) suffered pelvic lymph nodes recurrence. While 15 of 328 patients (4.5%) receiving ≥60Gy irradiation to PLNM experienced pelvic lymph bodes failure. No significant difference was identified between < 60Gy group and ≥60Gy groups regarding pelvic lymph nodes failure (P=0.753, chi-square test).

For patients with distant metastasis, lung was the primary site of metastasis (43/502, 8.6%), followed by para-aortic lymph node (21/502, 4.2%), cervical or supraclavicular lymph node (17/502, 3.4%), mediastinal lymph node (13/502, 2.6%), bone (14/502, 2.8%), liver (9/502, 1.8%) and other rare sites.

### Toxicity

The acute and chronic treatment related toxicities were shown in Table 3 and Table 4. No treatment related death was observed in our study. Some valuable data were missed because of the retrospective nature, 437 patients were available for assessing the acute hematologic (HM) toxicity, the incidences of acute grade 3 or greater HM toxicity was 57.9%. Only 335 patients were available for evaluating the acute gastrointestinal (GI) and genitourinary (GU) toxicities. The incidences of grade 3-4 nausea, vomit, abdominal pain, diarrhea and proctitis were 6.9%, 7.8%, 0.9%, 9.5% and 0.6%, respectively. All kinds of gastrointestinal toxicities were well controlled. Frequent micturition was recorded in 135 patients (40.3%) including 81 patients (24.2%) with grade 1 and 54 patients (16.1%) with grade 2. Other acute GU toxicities were not well recorded in our database.

A total of 369 patients were available for evaluating chronic toxicities, and 101 patients (27.7%) experienced chronic toxicities. Most of the chronic toxicities were mild to moderate (grade 1-2), accounting for 84.2% of all toxicities (85/101). As for severe toxicities, the incidences of grade 3-4 chronic GI and GU toxicities were 2.7 % (10/365) and 0.8% (3/365), retrospectively. Two patients (0.5%) developed rectovaginal fistula, while vesicovaginal fistula occurred in one patient (0.3%).

### Prognosis

We chose age, pathology (Scc vs Non-Scc), differentiation (high vs moderate vs low), T stage (T1 vs T2 vs T3), Number of PLNM (1 vs ≥ 2), common iliac LNM (no vs yes), regional LNM (single region vs multi regions), cycles of chemotherapy (≥ 4 vs 1-3 vs 0) as potential prognostic factors for patients with stage IIIC1 cervical cancer. After univariate analysis, T stage, number of PLNM, common iliac LNM, cycles of chemotherapy were significantly associated with OS. T stage, number of PLNM and cycles of chemotherapy significantly affected DFS. LC was greatly influenced by T stage. LRC was impacted by both number of PLNM and cycles of chemotherapy.

**Figure 3:**
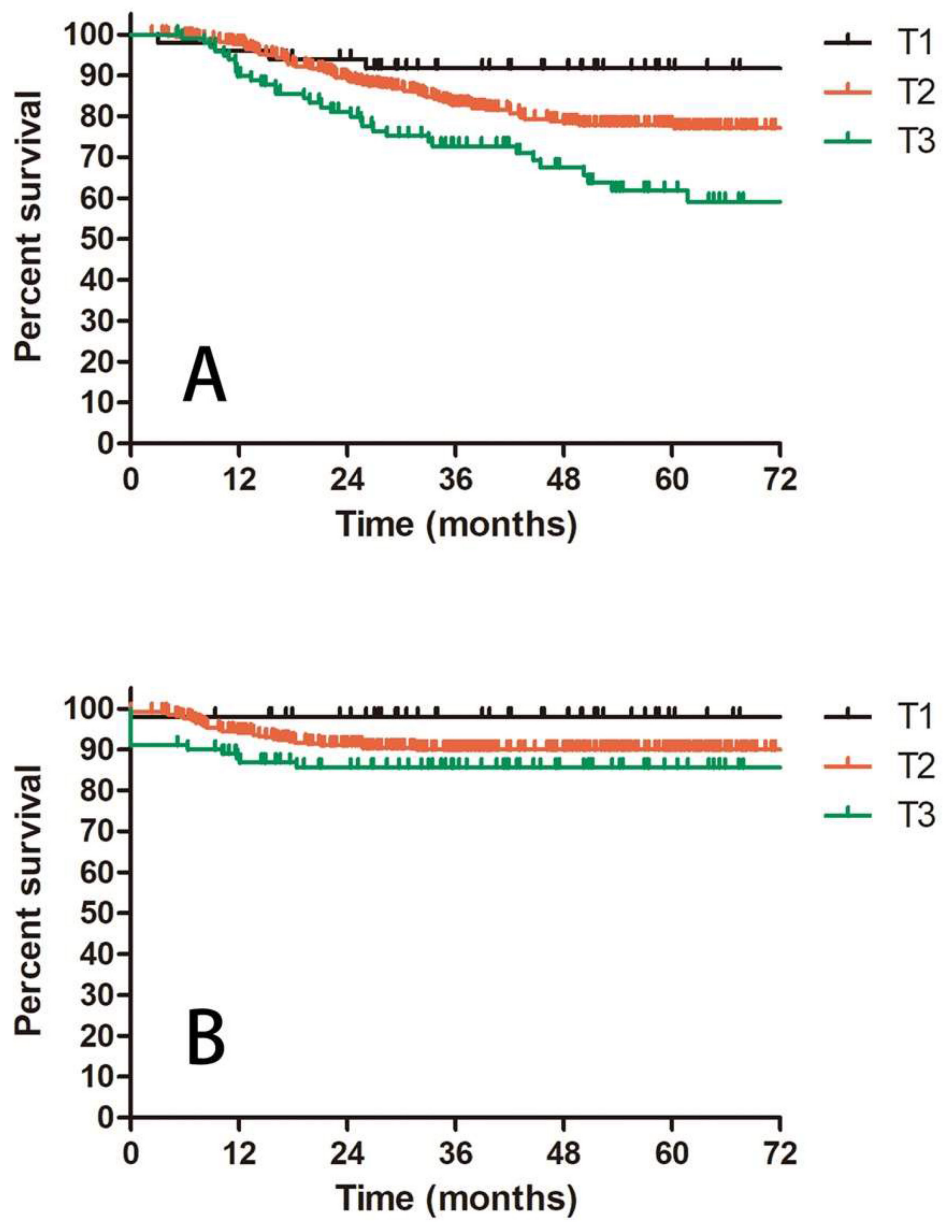
A: OS for patients with IIIC1 cervical cancer regarding different T stage; B: LC for patients with IIIC1 cervical cancer regarding different T stage.

**Figure 4:**
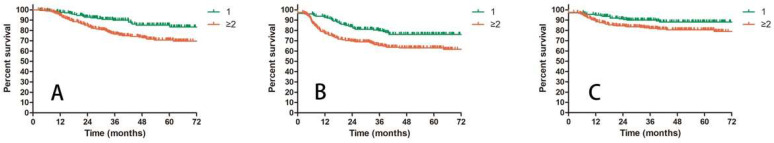
A: OS for patients with 1 or ≥2 positive pelvic lymph nodes; B: DFS for patients with 1 or ≥2 positive pelvic lymph nodes; C: LRC for patients with 1 or ≥2 positive pelvic lymph nodes.

**Figure 5:**
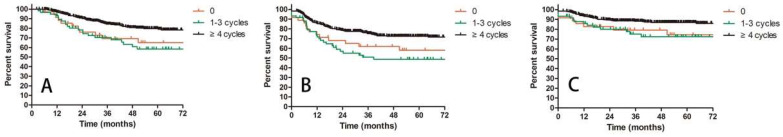
A: OS for patients receiving different cycles of concurrent chemotherapy; B: DFS for patients receiving different cycles of concurrent chemotherapy; C: LRC for patients receiving different cycles of concurrent chemotherapy.

Prognostic factors confirmed by univariate analysis were further involved in multivariate analysis. T stage, number of PLNM and cycles of chemotherapy remained correlated with survival outcomes. Advanced T stage was associated with worse OS and LC. The 3-year OS and LC for patients with T1, T2 and T3 stage were 91.8% vs 82.8% vs 72.6% (T1 vs T2 P = 0.109, T1 vs T3 P = 0.023, T2 vs T3 P = 0.056, Figure 3A) and 98.0% vs 90.0% vs 85.6% (T1 vs T2 P = 0.117, T1 vs T3 P = 0.048, T2 vs T3 P = 0.048, Figure 3B), respectively. Patients with two or more positive lymph nodes had nearly two-fold risk of death (HR = 1.892, 95%CI: 1.892-3.055, P = 0.009, Figure 4A), disease relapse (HR = 1.817, 95%CI: 1.246-2.651, P = 0.002, Figure 4B) and pelvic failure (HR = 1.810, 95%CI: 1.067-3.072, P = 0.028, Figure 4C) than those with only one positive lymph node. For patients receiving 0, 1-3, and ≥ 4 cycles of chemotherapy, the 3-year OS, DFS and LRC were 69.4% vs 70.4% vs 84.4% (Figure 5A), 61.9% vs 50.9% vs 75.1% (Figure 5B) and 79.2% vs 75.1% vs 88.2% (Figure 5C). Obviously, more cycles of chemotherapy (≥ 4 cycles) was associated with better OS, DFS and LRC. The detailed information of univariate and multivariate analyses was shown in Table 5 and Table 6.

## Discussion

The FIGO 2018 staging system of cervical cancer defined patients with positive pelvic lymph nodes as stage IIIC1 5. In the present study, we reported our experience in treating patients with IIIC1 cervical cancer with IG-IMRT. The 3-year OS, DFS, LC and LRC were 81.7%, 71.4%, 89.9% and 86.1%, respectively. These outcomes were quite comparable to the historic reports. The study design of Dang and colleagues was similar with ours 12, 40 patients with PLNM receiving definitive CCRT were enrolled in their study. The irradiation dose to positive lymph node was escalated to 62.5Gy in 25 fractions with SIB. The reported 3-year OS, DFS and LC were very excellent with 82.5%, 82.5% and 90.0%, respectively. However, the small sample size limited the persuasive power of this study. The 5-year CSS for IIIC1 cervical cancer was 62.1% in SEER database (including 6888 patients) 13. The reported 5-year OS was 61.9% for 4451 patients with NCDB 14. The results based on two national databases were obviously inferior than ours with a 5-year OS of 75.5%. In the SEER study 13, nearly 20% of patients were treated before 2000 when the standard treatment for cervical cancer was not established, and many advanced technologies were not applied in clinical use. The treatment modalities also varied significantly among different centers. The NCDB study did not clarify the treatment methods specifically 14. When conducting IMRT, clinicians should be pay enough attention to organ motion, Changes in the positions of related organs would lead to missing important target volumes and increasing dose to the OARs, which would further compromise clinical outcomes and increase toxicities. For patients with FIGO IIIC1 cervical cancer, the primary tumor, positive lymph nodes, cervix, uterus, rectum and bladder are the relevant target volumes and OARs. The fulfilment of bladder and rectum although significantly affect the position of cervix and uterus 16. With the help of IGRT, clinicians could make sure that the prescribed dose would be delivered to the right target volume, while the OARs would be avoided. In our study, all patients received definitive CCRT with IG-IMRT. The standard treatment and advanced technology might contribute to our excellent survival outcomes.

For stage IIIC1 patients with early T stage. Although both definitive surgery plus adjuvant CCRT and definitive CCRT were recommended by guidelines 7, most centers still prefer to perform surgery and other adjuvant treatment. In the study of GOG 109 17, 127 patients with T1a2, T1b and T2a received surgery and adjuvant CCRT, among which 110 patients had histological proven PLNM. The reported 4-year PFS and OS were 80% and 81%. In another study, 31 patients with histological confirmed PLNM after definitive surgery received adjuvant CCRT and consolidation chemotherapy, IMRT was adopted in this study. The estimated 3-year PFS and OS were 88.5% and 93.8%. Comparing these two studies with ours, the GOG 109 was conducted with 3D conformal radiotherapy (3D-CRT), our survival outcomes were obviously much better with a 3-year OS of 91.8% for T1 patients. The second study used IMRT when performing radiotherapy, consolidation chemotherapy was also involved, the 3-year OS was very comparable to ours (93.8% vs 91.8%). Landoni and colleagues 18 were the first to compare efficacy and toxicity between surgery and radiotherapy in patients with early-stage cervical cancer. They revealed that definitive radiotherapy was not inferior than surgery regarding clinical outcomes, fewer severe morbidities were observed in the radiotherapy group (12% vs 28%, P=0.0004). This study also established the role of definitive radiotherapy in the treatment of early stage cervical cancer. So, definitive CCRT is an effective alternative for stage IIIC1 cervical cancer with early T stage.

Significant relationship has been identified between irradiation dose and lymph nodes control rate. In a retrospective study 11, nine of 16 patients with PLNM receiving ≤ 58Gy irradiation experienced lymph node recurrence, while none of 21 patients with > 58Gy irradiation suffered lymph node failure. The application of SIB which can increase the BED of PLNM was associated with better lymph node control 10. Kim et al treated IIIC1 cervical cancer patients with a median irradiation dose of 62.6Gy (EQD2, α/β = 10) to positive lymph nodes with helical tomotherapy, the initial complete response (CR), partial response (PR) and stable disease (SD) were observed in 54, 2 and 2 lymph nodes 19. In the study of Bacorro et al 10, a total of 108 patients with 254 positive lymph nodes were identified, the mean total dose were 55.3Gy±5.6Gy (EQD2, α/β = 10), 23 patients suffered nodal recurrence (9.1%). 62.5Gy in 25 fractions (EQD2 = 65Gy, α/β = 10) were prescribed to positive lymph nodes in Dang's study 12, they enrolled 74 patients in total. No lymph nodes failure was observed with a median follow-up of 36 months. In our study, the median dose to PLNM was 60.2Gy in 28 fractions (EQD2 = 61Gy, α/β = 10) and the minimum dose was 56Gy in 28 fractions, nodal recurrence occurred in 21 patients (4.2%). No significant difference was found between < 60Gy group and ≥ 60Gy group regarding nodal relapse rate. Compared with the historical data, we achieved an excellent pelvic lymph node control rate. Although the size of positive lymph node also influenced the nodal control rate 10, our results based on 502 patients with IIIC1 cervical cancer indicated that a dose of 60Gy might be sufficient for controlling most positive lymph nodes.

Dose escalation radiotherapy can also increase dose to adjacent organs at risk (OARs). Therefore, the corresponding toxicities should be paid more attention. In our institute, IGRT was adopted when performing IMRT with SIB. With its help, we could guarantee that the prescribed dose was delivered to the right target. If the organ movement was so much that the target volume could not be well covered by PCTV or PGTV, we would immediately redesign the radiotherapy plan 15. With the help of all these advanced techniques, the present study reported quite tolerable treatment related toxicities. The incidence of acute grade 3-4 HM toxicity was 57.9%. All kinds of acute GI toxicities were well controlled within 10%. The incidences of grade 3-4 chronic GI and GU toxicities were 2.7 % and 0.8%, respectively. Dang and colleagues reported grade 3 acute GI toxicity rate of 8.1% and grade 3-4 acute HM toxicity rate of 44.4% with IMRT based SIB 12. In the RetroEMBRACE study including 731 patients, the actuarial 5-year grade 3-5 morbidity was 5% and 7% for bladder and gastrointestinal tract, respectively 20.

Distant metastasis was the major failure pattern in the current study, accounting for 66.9% (101/151) of disease failures. So, additional systemic treatment might be of great value for this group of patients. In a retrospective study from Japan 21, patients with positive lymph nodes who received systemic chemotherapy after surgery experienced fewer distant failures than those who did not (19.2% vs 24.6%, P < 0.001). Since adjuvant chemotherapy was not routinely used in our institute, and only 17 patients underwent adjuvant chemotherapy. We could not evaluate the efficacy of adjuvant chemotherapy at present. Further prospective study should be allocated.

After the FIGO 2018 staging system of cervical cancer was published, Matsuo, et al conducted a validation study with records from SEER database 13. They illustrated that, patients with stage IIIC1 had better survival outcomes than those with stage IIIA and IIIB. The 5-year CSS was significantly affected by T stage varying from 39.3% to 74.8%. The present study also revealed the significant relationship between T stage and survival outcomes (OS, LC). Except for T stage, several studies indicated that the characteristics of positive lymph nodes including number, size and volume also impacted the prognosis of cervical cancer 1, 2, 22. In our study, number of PLNM was finally confirmed as prognostic factors for OS, DFS and LRC in patients with IIIC1 cervical cancer. For patients with one positive lymph node and ≥ 2 positive lymph nodes, the 3-year OS, DFS and LRC were 90% vs 76.4% (P = 0.009), 79.6% vs 65.2% (P = 0.002) and 90% vs 81.9% (P = 0.028), respectively. All of these indicated the heterogeneity of stage IIIC1 cervical cancer. Further stratification might be valuable for this group of patients. The pattern of lymph nodes metastasis in cervical cancer is “station followed by station”, which means from one station to another higher station with rare skipping metastasis 23. We divided patients into two groups (single region and multi regions) based on the distribution of lymph nodes. However, we failed to show any relationship between the distribution of lymph nodes and survivals.

Another meaningful finding of this study is that more chemotherapy cycles brought better survivals. Patients receiving ≥ 4 cycles of chemotherapy displayed the highest OS, DFS and LRC compared with those who had no chemotherapy or 1-3 chemotherapy cycles. The 3-year OS was 84.4% for patients with ≥ 4 chemotherapy cycles, while it was only about 70% for patients with 0-3 cycles. However, the cycles of chemotherapy had no impact on LC, suggesting that the effect of chemotherapy was more about systemic control. The better systemic control would be further converted into survival benefits. Schmid, et al also investigated the relationship between cycles of chemotherapy and survivals. They found that more chemotherapy cycles (5-6 cycles vs 0-4 cycles) reduced the incidence of distant metastasis for patients with positive lymph nodes. Therefore, patients with IIIC1 cervical cancer should receive adequate cycles of concurrent chemotherapy.

Many studies regarding IG-IMRT in cervical cancer have been published in recent years. However, most of them focused on the clinical outcomes and toxicities. Wang's research revealed that IG-IMRT brought excellent clinical outcomes and accepted toxicities. the 3-year OS, DFS and LC for locally advanced cervical cancer were 83.0%, 75.0% and 87.4%, respectively, the the incidence of G3+ chronic gastrointestinal and genitourinary toxicities were 2.3% and 1.3% 15. Chopra S' study and Grabenbauer GG's study proved the advantages of IG-IMRT than 3D-CRT regarding toxicities 24, 25. Fröhlich G's study 26 revealed that the image-guided adaptive interstitial brachytherapy had dosimetric advantages compared with conventional BT. Just as the above studies, we also found that the IG-IMRT was well tolerated with excellent survivals. While, another two meaningful conclusions were also identified. First, T stage and number of PLNM significantly affected the survivals indicating the heterogeneity of IIIC1 cervical cancer patients. Second, Adequate chemotherapy (≥ 4 cycles) should be administered in this group of patients as much as possible. All of these findings were of great significance in clinical practice.

Several limitations still existed in our study. Firstly, the retrospective nature resulted in the loss of important medical records. The detailed information of PLNM characteristics of 40 patients were missing. Secondly, this study enrolled patients from a single center. Although we drew several valuable conclusions, validation with patients from other institutes was still needed.

## Conclusion

IG-IMRT was well tolerated with excellent survival outcomes in patients with FIGO IIIC1 cervical cancer. A dose of 60Gy was sufficient for controlling most positive lymph nodes. T stage and number of PLNM significantly influenced the survivals indicating the heterogeneity of stage IIIC1 cervical cancer patients, which should be further stratified. Adequate chemotherapy (≥ 4 cycles) should be administered in this group of patients as much as possible.

## Figures and Tables

**Table 1:** Characteristics of patients with FIGO IIIC1 cervical cancer

Characteristics	No. (%)
Age	
Median 50 (range, 26 - 78)	
Pathology	
Squamous cell carcinoma	471 (93.8%)
Adenocarcinoma	23 (4.6%)
Adenosquamous carcinoma	5 (1.0%)
Others	3 (0.6%)
Differentiation	
High	21 (4.2%)
Moderate	99 (19.7%)
Low	59 (11.8%)
Undefined	323 (64.3%)
Primary tumor stage	
T1	50 (10.0%)
T2	352 (70.1%)
T3	100 (19.9%)
Number of PLNM	
1	177 (35.2%)
≥2	285 (56.8%)
Unclear	40 (8.0%)
Common iliac LNM	
Yes	88 (17.5%)
No	374 (74.5%)
Unclear	40 (8.0%)
Regional LNM	
Single region	321 (63.9%)
Multi regions	141 (28.1%)
Unclear	40 (8.0%)
Dose to PLNM	
Median 60.2 Gy	
< 60 Gy	134 (26.7%)
≥60 Gy	328 (65.3%)
Unclear	40 (8.0%)
Cycles of concurrent chemotherapy	
0	36 (7.2%)
1-3	59 (11.7%)
≥4	407 (81.1%)
Adjuvant chemotherapy	
Yes	17 (3.4%)
No	485 (96.6%)

Abbreviations: PLNM = pelvic lymph node metastasis, LNM = lymph node metastasis

**Table 2:** Patterns of disease recurrence

Patterns of failure	No. (%)
Pelvic recurrence	68 (13.5%)
Cervix	19 (3.8%)
Persist disease	13 (2.6%)
Pelvic lymph nodes	21 (4.2%)
Parametrium	8 (1.6%)
Uterus	4 (0.8%)
Vagina	4 (0.8%)
Others	6 (1.2%)
Distant metastasis	101 (20.1%)
Lung	43 (8.6%)
Bone	14 (2.8%)
Liver	9 (1.8%)
Brain	1 (0.2%)
Para-aortic lymph nodes	21 (4.2%)
Mediastinal lymph nodes	13 (2.6%)
Cervical or supraclavicular lymph nodes	17 (3.4%)
Inguinal lymph nodes	4 (0.8%)
Others	7 (1.4%)
Total	151 (30.1%)

Note: some patients experienced more than one kind of disease recurrence at the same time.

**Table 3:** Acute treatment related toxicities

Toxicity	Grade 1	Grade 2	Grade 3	Grade 4
Fatigue ^a^	161 (48.1%)	37 (11.1%)	11 (3.3%)	—
Hematologic ^b^	24 (5.5%)	135 (30.9%)	234 (53.5%)	19 (4.4%)
Nausea^ a^	155 (46.3%)	52 (15.5%)	23 (6.9%)	—
Vomit^ a^	56 (16.7%)	67 (20.0%)	26 (7.8%)	0
Abdominal pain^ a^	123 (36.7%)	21 (6.3%)	3 (0.9%)	—
Diarrhea^ a^	117 (34.9%)	75 (22.4%)	32 (9.5%)	0
Proctitis^ a^	43 (12.8%)	15 (4.5%)	2 (0.6%)	0
Frequent micturition^ a^	81 (24.2%)	54 (16.1%)	—	—

Notes: a, 335 patients were available for assessing toxicity; b, 437 patients were available for assessing toxicity

**Table 4:** Chronic treatment related toxicities^ a^

Toxicity	Grade 1	Grade 2	Grade 3	Grade 4
Gastrointestinal^ b^	14 (3.8%)	27 (7.4%)	8 (2.2%)	2 (0.5%)
Diarrhea	7 (1.9%)	0	0	0
Proctitis	7 (1.9%)	10 (2.7%)	5 (1.4%)	1 (0.3%)
Ileus	0	0	4 (1.1%)	0
Intestinal perforation	0	0	1 (0.3%)	1 (0.3%)
Genitourinary^ c^	31 (8.5%)	16 (4.4%)	3 (0.8%)	0
Frequent micturition	6 (1.6%)	2 (0.5%)	—	—
Urinary incontinence	23 (6.3%)	2 (0.5%)	0	—
Cystitis	3 (0.8%)	13 (3.6%)	3 (0.8%)	0
Rectovaginal fistula	0	0	2 (0.5%)	0
Vesicovaginal fistula	0	0	1 (0.3%)	0
Total	46 (12.6%)	39 (10.7%)	14 (3.8%)	2 (0.5%)

Notes: a, 365 patients were available for assessing toxicity; b, some patients suffered more than one kind of gastrointestinal toxicity; c, some patients suffered more than one kind of genitourinary toxicity.

**Table 5:** Univariate analysis for stage IIIC1 patients regarding OS, DFS, LC, LRC.

Variables	OS		DFS		LC		LRC	
	HR (95% CI)	P	HR (95% CI)	P	HR (95% CI)	P	HR (95% CI)	P
Age	1.015 (0.993-1.038)	0.177	0.991 (0.974-1.009)	0.335	1.027 (0.995-1.060)	0.098	1.004 (0.978-1.030)	0.782
Pathology								
Scc	1		1		1		1	
Non-Scc	0.779 (0.317-1.914)	0.586	1.346 (0.746-2.428)	0.324	0.045 (0-7.749)	0.238	0.436 (0.107-1.777)	0.247
Differentiation								
High	1		1		1		1	
Moderate	1.015 (0.343-3.002)	0.978	0.719 (0.325-1.588)	0.414	0.868 (0.245-3.076)	0.826	0.849 (0.284-2.539)	0.769
Low	1.729 (0.581-5.144)	0.325	0.992 (0.437-2.255)	0.985	0.749 (0.187-2.995)	0.683	0.744 (0.224-2.471)	0.629
Primary tumor stage								
T1	1		1		1		1	
T2	2.413 (0.880-6.619)	0.087	1.344 (0.721-2.506)	0.352	4.902 (0.670-35.839)	0.117	2.589 (0.809-8.292)	0.109
T3	4.664 (1.652-13.166)	**0.004**	2.177 (1.117-4.244)	**0.022**	7.777 (1.022-59.148)	**0.048**	2.995 (0.873-10.281)	0.081
Number of PLNM								
1	1		1		1		1	
≥2	2.115 (1.321-3.385)	**0.002**	1.874 (1.289-2.723)	**0.001**	1.549 (0.829-2.895)	0.170	1.799 (1.060-3.051)	**0.029**
Common iliac LNM								
No	1		1		1		1	
Yes	1.759 (1.119-2.766)	**0.014**	1.385 (0.932-2.056)	0.107	0.743 (0.333-1.658)	0.468	1.129 (0.628-2.030)	0.686
Regional LNM								
Single region	1		1		1		1	
Multi regions	1.228 (.804-1.876)	0.342	1.149 (0.805-1.640)	0.444	0.699 (0.356-1.373)	0.298	1.019 (0.610-1.701)	0.944
Cycles of chemotherapy								
≥4	1		1		1		1	
1-3	2.059 (1.116-3.798)	**0.021**	1.715 (0.998-2.946)	**0.051**	2.245 (0.940-5.358)	0.069	2.009 (0.951-4.244)	0.067
0	2.291 (1.408-3.729)	**0.001**	2.145 (1.415-3.251)	**<0.001**	2.004 (0.978-4.273)	0.057	2.196 (1.213-3.979)	**0.009**

Abbreviations: HR = hazard ratio, CI = confidence interval, Scc = squamous cell carcinoma, PLNM = pelvic lymph nodes metastasis, LNM = lymph nodes metastasis.

**Table 6:** Multivariate analysis for stage IIIC1 patients regarding OS, DFS, LC, LRC.

Variables	OS		DFS		LC		LRC	
	HR (95% CI)	P	HR (95% CI)	P	HR (95% CI)	P	HR (95% CI)	P
Primary tumor stage								
T1	1		1		1		—	
T2	2.593 (0.808-8.318)	0.109	1.273 (0.660-2.456)	0.471	4.902 (0.067-35.839)	0.117		
T3	4.027 (1.212-13.382)	**0.023**	1.710 (0.837-3.493)	0.141	7.777 (1.022-59.148)	**0.048**		
Cycles of chemotherapy								
≥4	1		1		1		1	
1-3	2.360 (1.205-4.622)	**0.012**	1.795 (0.963-3.348)	0.066	2.088 (0.873-4.994)	0.098	2.437 (1.102-5.387	**0.028**
0	2.405 (1.437-4.024)	**0.001**	2.315 (1.501-3.568)	**<0.001**	1.971 (0.937-4.146)	0.074	2.446 (1.348-4.438)	**0.003**
Number of PLNM					—			
1	1		1				1	
≥2	1.892 (1.172-3.055)	**0.009**	1.817 (1.246-2.651)	**0.002**			1.810 (1.067-3.072)	**0.028**
Common iliac LNM			—		—		—	
No	1							
Yes	1.364 (0.859-2.164)	0.188						

Abbreviations: HR = hazard ratio, CI = confidence interval, PLNM = pelvic lymph nodes metastasis, LNM = lymph nodes metastasis
